# Medical record linkage in health information systems by approximate string matching and clustering

**DOI:** 10.1186/1472-6947-5-32

**Published:** 2005-10-11

**Authors:** Erik A Sauleau, Jean-Philippe Paumier, Antoine Buemi

**Affiliations:** 1 Service des études et applications de l'information médicale (SEAIM), Hospital, 87 Ave d'Altkirch, F68051 Mulhouse, France

## Abstract

**Background:**

Multiplication of data sources within heterogeneous healthcare information systems always results in redundant information, split among multiple databases. Our objective is to detect exact and approximate duplicates within identity records, in order to attain a better quality of information and to permit cross-linkage among stand-alone and clustered databases. Furthermore, we need to assist human decision making, by computing a value reflecting identity proximity.

**Methods:**

The proposed method is in three steps. The first step is to standardise and to index elementary identity fields, using blocking variables, in order to speed up information analysis. The second is to match similar pair records, relying on a global similarity value taken from the Porter-Jaro-Winkler algorithm. And the third is to create clusters of coherent related records, using graph drawing, agglomerative clustering methods and partitioning methods.

**Results:**

The batch analysis of 300,000 "supposedly" distinct identities isolates 240,000 true unique records, 24,000 duplicates (clusters composed of 2 records) and 3,000 clusters whose size is greater than or equal to 3 records.

**Conclusion:**

Duplicate-free databases, used in conjunction with relevant indexes and similarity values, allow immediate (i.e.: real-time) proximity detection when inserting a new identity.

## Background

Because of the fast growth of communication protocols (Internet technologies amongst them), health services are undergoing a large paradigm change: a shift from institution-centred care to consumer-centred care. Unambiguous identification of patients is a critical success factor for health care reform and for the provision of speedy, safe, high quality, comprehensive and efficient health care. We may consider more complete information on which to base potentially life-critical clinical decisions and less wasted time and less inconvenience as a result of hunting for information and/or re-gathering as being amongst the benefits of positive identification. However, client information flows are often substantially limited by an inability to positively identify the subjects of care and to locate their relevant details amongst an extensive array of data repositories which may be unlinked, duplicated and catalogued in different ways. Health workers are now confronted with "health records" for each patient dealing with contacts in a hospital, with a general practitioner, etc. If every contact with the patient is an opportunity to collect information, the information noted must, within an identification domain, be perfectly linked to the patient and identified within the domain by a unique identification number (**UID**) and a minimum profile (i.e. a set of features such as name, first name, gender and date of birth). This UID and profile then constitute the identity of the subject. When matching different identification domains, profiles are compared in order to track the same individuals. The ultimate aim is a complete "data reconciliation", meaning the coherent association between a unique identity and several other features like medical data, demographic data, ... The expression "record linkage" refers to the use of an algorithm technique to match records from different datasets which correspond to the same statistical unit [1,2]. But whatever the chosen method is, the final decision remains in the hands of a human specialist. Computer logic, based upon Boolean operations can basically tell us if two values are equal ("True or False", "1 or 0"), but it is limited if the answer has to be a bit more fuzzy. The decision-making ability of the human mind is still difficult and time consuming to replicate.

Medical information, and by extension identity records, has to be over-time stable, mainly due to legal requirements and for reasons of traceability. So we must be sure, until a final decision is made by the specialist, not to alter the original data in any way during the linkage process. It would be useful, therefore, when comparing records, to process atomic parts of them (such as name, first name, date of birth ...) in order to avoid the most common mistakes made during data entry. This step, called standardization, will ensure a greater effectiveness of the comparison algorithm. Most of the mistakes are a result of not adhering to data entry guidelines (i.e. abbreviations, accented letters, date format...), which are too often the responsibility of the operator. Some others appear when attempting to match records from different data sources (or different identification domains), ruled by heterogeneous and non-compatible data-entry guidelines. This standardization step is mandatory and its lifetime should not exceed the comparison operation.

In a database containing *n *records, record linkage is often described as an O(*n*^2^) complexity problem, due to its Cartesian aspect. The brute force approach, comparing each record to every other record, requires (*n*^2^-*n*)/2 comparisons. This approach, while being the most reliable (as no record is missed during the comparison process) is also the most time consuming and least effective (CPU load speaking). To reduce the number of comparisons, indexation of databases by blocking techniques is used. The data sets are split into smaller blocks and only records within the same blocks are compared.

Whichever blocking technique is chosen, the principle of a record linkage process is to try to pair similar records. But how is the decision which qualifies two different records as "duplicates" made? A basic way is to compare each atomic part of a sample record (i.e. the fields) to its counterpart in the reference record. If a binary equality is obtained, we can then assume that the entities are the same. The main drawback with such a method is that keystroke mistakes or subtle spelling changes are ignored, even after standardization. To be more efficient, we must enhance the comparison principle. While a binary operator returns a Boolean value (1 if the fields are strictly the same, 0 if they are different), a similarity operator will return a score ranging from 0 to 1, showing fields proximity and quantifying their differences. The higher the result, the nearer the field values are. Such a similarity operator is based on approximate string matching methods, so non-literal data fields such as dates or numbers have to be converted before processing. The main approach of approximate string matching has always, until now, been based on the edit-distance [3,4], the oldest kind of algorithm according to Navarro [5,6]. For example, Levenhstein's distance [7] is the minimum number of operations on individual characters like substitutions, insertions and deletions, needed to transform one string to another. It remains the most flexible although it is no longer the most effective, at least in "text retrieval" cases. The Smith-Waterman algorithm [8] is one of the most popular algorithms for edit-distance. Much of its power is due to its ability to introduce gaps in the records (sequence of non-matching symbols). Hence, the use of such an algorithm on each field of records allows us to calculate the same number of atomic similarities (noted **aS **in the sequel) linking the fields of the sample and the reference records. Such atomic values are then combined in a weighted mean to obtain a record to record similarity or global similarity (**gS**) value. If one uses k fields for comparing records then  where **w**_j _are the weights (corrected so that the sum of all of them is 1) in the mean. Indeed, not all the atomic similarities have the same discriminative power when comparing two records. For example, gender is less reliable than date of birth: two records with the same gender have less chance of representing the same identity than two records with the same date of birth. The weights are obtained, for example, by the Expectation-Maximization algorithm [9,10]. Several general algorithms for record linkage have been written since the 70's, most of them in biomedical papers in order to perform epidemiological studies [11]. The seminal theoretical paper on record linkage by Felligi and Sunter [12] has, as its goal, the division of record pairs into linked pairs (designated matches), possible linked pairs (pairs for which human oversight, also known as clerical review, is needed) and non-linked pairs (non-matches). Their classification rule is based on the comparison of the common fields of the two sets of records. To be a little more specific, let us suppose that all the records of a dataset are distributed into two equal sets, say A and B, and that furthermore we create three more sets: for matched pairs, for non-matched pairs and a set of possible matched pairs. If each record has k fields, it is possible to define an agreement k-vector γ (all the atomic similarities for example) between a record of the set A and a record of the set B. The main issue of the Felligi-Sunter theory is to define an optimal linkage rule (for given levels of type I and type II errors), where optimality is defined as minimising the probability of classifying a pair in the set of possible matched pairs. Assuming conditional independence of the k components of γ, the decision rule is a function of  where those weights w_j _are . Here **m**(γ_j_) and **u**(γ_j_) are the conditional probability of observing γ_*j *_given that the pair is respectively a true matched pair and a true non-matched pair. For example, Jaro [13,14] uses the EM algorithm to estimate these weights (or at least the **m**(γ_j_)s).

Results provided by those techniques of record linkage consist of a list of paired records linked by a global similarity score. Whilst being a good starting point, such a presentation is far from being the most efficient for clerical review. Specialists have to deal with complex patterns of similarities between several records split-down into a list of similarities between couples of records. We can then try to build clusters of duplicates, "n-plicates". A weak definition of a cluster could be *"a set of entities which are alike and entities from different clusters are not alike"*. Methods for clustering can be divided into hierarchical, graph-based (equivalent to the graph partitioning), model-based or mixed methods [15,16]. An additional stage in the representation of the records with their similarities may be necessary. From a representation of records in clusters, we can switch to a representation of graphs. Following the graph theory, the clusters become undirected sub-graphs with vertices (records) and edges (global similarity value). It would be useful, for the visual comfort of the user, to represent these clusters in a reliable manner with vertices and edges, whose lengths are proportional to the strength of the relation between the vertices (global similarity). The most used methods for graph drawing are force-directed methods [17-19]. Generally, these methods view a graph as a system of particles (vertices) with forces acting between them (edges). They seek a state of balance where the sum of the forces acting on each particle is zero. All these methods are relatively simple to implement, heuristic improvements can easily be added, but they can be time consuming.

**Figure 1:**
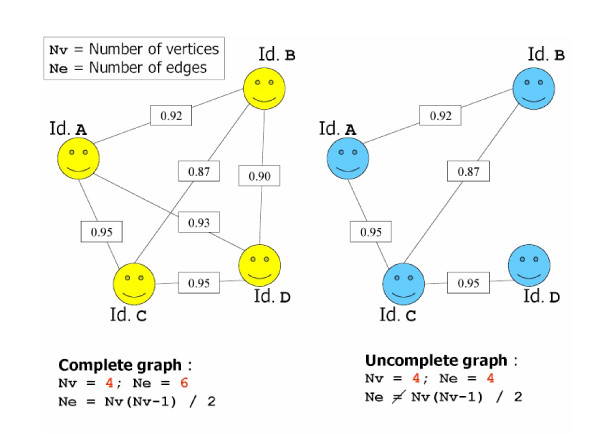
Example of a complete graph (left panel) and of an incomplete graph (right panel).

But specialists also have to deal with the possibility that some of the relations between some record pairs may be unreliable. For example, two different paths between two records can exist: one path with only edges labelled "duplicate" and one with at least one edge labelled "non-duplicate". Hence, some of the sub-graphs are complete (each of the vertices is linked with each other in the sub-graph by an edge), like the left panel on Figure 1. But some are incomplete (n vertices but fewer than n(n-1)/2 edges), like the right panel on Figure 1. If we choose not to enforce transitivity of the relation "is a duplicate of", unlike some other authors [20,21], the vision of this sub-graph as a "n-plicate" is not so straightforward. Thus, it would be interesting to consider complete sub-graphs within this incomplete one, and then to consider each of these last sub-graphs as "n-plicate". Methods for this result are derived from graphs partitioning algorithms [22], in an unsupervised situation (the number of sub-graphs is, *a priori*, unknown). They go on to delete several edges in order to isolate groups of vertices. Such a cut is said to be of minimum weight, if the sum of the weights on the edges is the minimum necessary to isolate such groups of vertices. The gain of a vertex is the difference between the sum of the similarities with vertices of other sub-graphs and the sum of the similarities with vertices of its own sub-graph. Notably, the gain is the cost when a vertex changes its sub-graph.

Our very general aim is to provide specialists with a reliable presentation of a set of several potentially duplicated records in a data set with a score showing how similar records in this homogeneous set are. These specialists may be responsible for merging similar records or for avoiding duplicate entry. This method, then, deals with two different situations: batch and real-time data browsing.

## Methods

The method used for achieving our goal comprises three steps: the first one is a pre-processing of data in order to eliminate the most common errors and to present this data in a convenient way; the second is to compare records and the third is to organize the records. We will focus on showing its application upon a single data source, even if the proposed technique can be applied to link foreign domain records (i.e. a sample data source to a reference one).

A particularity in Anglo-Saxon countries and in France (but not in all Latin countries) is the use by a married woman of her husband's name: her birth name is no longer used but "replaced" by her married name. Because of the instability of this married name (divorced persons), the birth name is always recorded in a patient database to guarantee an over-time reference. But a high risk of errors exists while encoding names between birth name and married name. First of all, we define a typical patient-identity record as constituting a unique identification number and a minimum profile: birth name, married name, first name, gender and date of birth. We are working, here, on the patient database of our hospital as it was on the 1 st July 2003. This paper reports the use of our proposed method on this database from which we want to eradicate duplicates. Each of the variables belonging to the patient profile is already encoded in formatted fields within the database and thus does not need any parsing or re-allocating.

Accents, hyphens and apostrophes may be uncommon in English, but are very important and may appear often in French or in other Latin languages. Data entry of patient identities is, for the most part, under human control and subject to missing data or data entry errors (such as typographical or keystroke mistakes), non standard abbreviations or differences in detailed schemas of records from multiple databases. We first of all try to standardize the strings before comparing them with the algorithm: replacing all accented letters with the same unaccented letter, converting all strings in uppercase, replacing punctuation signs with a space, discarding all non-informative spaces (double spaces, spaces at the end of strings...), discarding all spaces in names and, in double first names (quite different from first name and initial in English), replacing the first part by its first letter (for example "Jean-Philippe" becomes "J PHILIPPE").

Baxter et al [23] describe four different blocking methods. The standard blocking method clusters records that share an identical blocking key, composed of one or more attributes of each record, like the postcode or phonetic encoding of names. The sorted neighbourhood method [24] sorts the records based on a sorting key and then moves a window of fixed size sequentially over the sorted records. Records within the window are then compared with each other. In the bigram indexing method [25], the blocking key values are converted into a list of bigrams, and sub-lists of all possible permutations are built using a threshold. The canopy clustering method [26] creates overlapping subsets, called "canopies" composed, for each record, of all the records within a certain loose threshold distance. For indexing the database, we chose a blocking method borrowed from this last technique. For each record a certain number of blocking keys is calculated, each one being three bytes in length which allow us to scan, for a given record, not the entire database but only the records whose blocking keys correspond. The structure of these keys is very simple. They are comprised of the first three characters of the standardized string (normal version) and the last three characters (inverted version). These two keys are computed for birth name (i.e. maiden name for a married woman), first name and, if it exists, married name. The use of inverted keys allows us to compare strings with an error on the first few characters, which seems to be common in patient databases. Furthermore, the speed of the algorithm remains high and the window screening remains feasible. Two records sharing the same canopy are compared if they meet one of the following two conditions:

• Their dates of birth and their gender are the same;

• Their gender are the same and their blocking keys on birth name and married name (if necessary) meet at least one of these two conditions:

     1. their normal or reversed blocking keys for birth names or for married names are the same

     2. the normal (respectively the reversed) blocking key for birth name in one of the two records is the same as the normal (respectively the reversed) blocking key for married name in the other record.

A comparison is made for each atomic part (i.e. field) of the identity record, giving an atomic similarity value for each. In order to compare two records, we run the approximate string matching algorithm on the different matching variables and write out six atomic similarities: three for the comparisons of the two dates of birth, the two first names and the two birth names, one, if necessary, between the married names (otherwise the atomic similarity is considered as missing), one if necessary between the married name of the first record and the birth name of the second (otherwise missing) and one, if necessary, between the birth name of the first record and the married name of the second (otherwise missing). From a series of different algorithms [27], we chose to use an algorithm we call the Porter-Jaro-Winkler algorithm [28], an improvement of an initial algorithm [13,14]. Its basic principle is to compare two strings *C*_*1 *_and *C*_*2*_, and to compute a similarity rate. It considers letters in the first string which are within half the length of the second string. In addition, some errors are penalized less harshly (visual scanning errors, keypunch errors and errors at the end of the string with respect to the errors at the beginning of the string). It is a looser matching criterion than edit-distance and, unlike the other techniques, it is not an "all or nothing" computation, but gives a degree of match. Noting *L*_*1 *_and *L*_*2*_, *C*_*1 *_and *C*_*2*_'s respective lengths, the similarity rate is obtained by , where *Nc *is the number of common characters in the two strings and *Nt *the number of transpositions. From this basic formula, several improvements are successively obtained, but we use only two of them:

• The number of "similar" characters *Ns *replaces the number of common characters *Nc*. A character is decreed "similar" to another if it belongs to one of 36 couples defined in the algorithm, which are for example (X, K), (5, S), (O, Q);

• Additional weight is given to the similarity when the first four characters of *C*_*1 *_and *C*_*2 *_are similar or identical. Typographical errors are often not located at the beginning of the string, but rather in its body;

According to the authors, a similarity of less than 0.7 means two different strings and more than 0.95 means two similar strings (common strings exhibit a rate of 1). We then add different refinements to this algorithm. We weight the atomic similarities of names (birth names and married names) depending on their frequency in the database: the more frequent a name is, the less the similarity seems to be credible. In this early report of our work, we have created just two categories according to the name frequency (approximately 5 per 10,000) and under-weight if necessary the similarity of 0.05. This 5 per 10,000 cut-off was decided from the graph of the names frequency, which has an inverse function aspect with the end of the strong decrease about 5 per 10,000. We also take into account the difficulty of comparing two strings of different lengths which present a strong similarity on the shorter length. For instance, the names "ABC" and "ABCDEFG" are quite different but have an atomic similarity according to the initial algorithm of 1. In this case, we calculate a similarity like the initial one minus 0.01 times the number of additional characters (4 in the above example, yielding an atomic similarity of 0.96). A global similarity is then computed as a weighted mean of atomic similarities. Because of the particular configurations of our records (with or without married names) we use a pragmatic weighting based on common weights used in literature and on our prior knowledge. Once again, we define a few rules:

1. If the two birth names and the two first names are almost the same (i.e.: aS > 0.7) then the date of birth should have a high discriminative weight;

2. If the similarity between the married name of a record and the birth name of a second record is more than the similarity between the two birth names then we suppose that an inversion between the married name and the birth name has occurred.

The weights are standardized to 1. A complete description of this weighting step appears in the Appendix. For example, the simplest situation (without any married name to compare) can be split into two sub-cases: if the birth name's atomic similarity is high (i.e. > 0.7) then the weight for birth names is 1/3, the weight for first names is 1/6, the weight for dates of birth is 1/2, otherwise the three weights are respectively set to 1/2, 1/4 and 1/4. Unlike the Felligi-Sunter probabilistic theory, we chose not to use two thresholds which would classify pairs into linked, non-linked and possibly-linked, but to determine a unique threshold with a set of non-linked pairs and a set of possibly-linked pairs. Before analyzing the database and after multiple experiments, this threshold was set to 0.85.

To achieve the clustering step, because of our confidence in the similarity algorithm, we need a simple, unsupervised (the number of clusters is, *a priori*, unknown) hard-clustering method (not a probability of classification). Our choice is a greedy agglomerative algorithm with complete linkage to avoid clusters in a chain. For graph drawing, we will choose a very simple version of a force directed algorithm, but in the early version of our work, this functionality is not yet implemented. Then, in order to automate the graph partitioning, several algorithms have been proposed [29-33], they are often heuristics based on seminal algorithms like Kerninghan-Lin or Fiduccia-Mattheyses. Because of our confidence in the previous steps and the use of the Porter-Jaro-Winkler algorithm, a simple algorithm based on the gain is then used to partition incomplete clusters in complete objects (clusters or isolated records).

## Results

With 300,859 records in our patient database, the brute force approach would need 45 10^9 ^comparisons. By using blocking variables, only 24.8 10^6 ^comparisons are computed, which means a 1,825-fold gain. In the database, there are 287,850 different canopies with a maximum canopy size of 11,550 records. In fact, the distribution of the size of the canopies is very left-skewed. If the mean size is 82 and the standard deviation 195, the median is 18 and the 95^th ^percentile is 398. The most frequent size is 7. Each record of the database resides (in average) in 8.9 canopies.

Among 300,859 records, our method detects 38,083 couples, whose global similarity equals or exceeds the decision threshold set to 0.85. The Figure 3 summarises the results of our entire method applied to this data set. Table 1 shows the number among these 38,083 couples with exact concordance in global similarity and in atomic similarities. This table shows that 9,566 couples exhibit a unit value for their global similarity. Even if they have different UIDs, these couples seem to correspond to 9,566 different individuals. The value of the atomic similarities for the fields birth name, first name and date of birth is exactly one in about 70% of the couples but less for married name. For the comparisons, when accurate, between birth name and married name, 3,065 couples match exactly (4%). This means that in 3,065 pairs the married name of a record is exactly the same as the birth name of the other record of the pair, probably indicating an inversion between these names. The Table 2 describes the characteristics of global similarity and atomic similarities among the remaining couples, without perfect concordance. About 29,000 couples exhibit a value of global similarity which is not exactly the unit 1 but a value with a mean (and a median) of 0.92. The distribution is approximately uniform on the interval [0.85 – 1.00], except for a small peak at 0.95 corresponding to the underweight of 0.05 concerning exact matching of records with frequent names. Concerning birth name, first name and date of birth, among about 13,000 remaining couples without a unit value of 1, the mean is about 0.80 but the quartiles show that the similarity on first name is more left-skewed than the similarity on birth name. The similarities between married names are globally higher than the similarities between birth names. Among the 16,013 comparisons remaining in the comparisons between birth name and married name, only about 800 exhibit a similarity higher than 0.75 and the mean of these 16,013 is 0.36.

**Figure 2:**
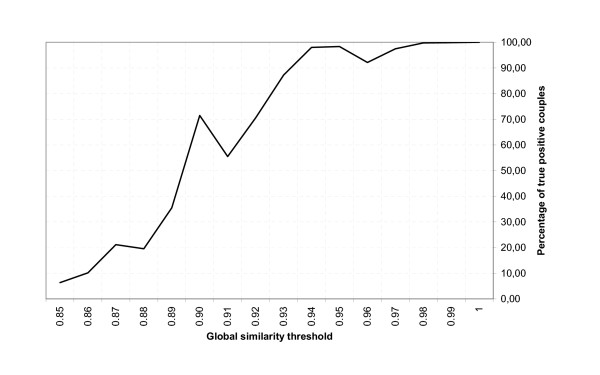
Percentage of true positive couples by global similarity threshold value.

**Figure 3:**
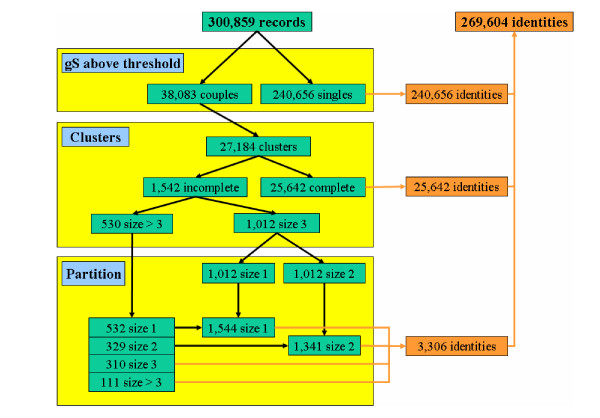
Summary of the entire linkage procedure.

**Table 1:** Number of exact concordance in global similarity (gS) and atomic similarities (aS) in the 38,083 identified pairs in a database of 300,859 patients

	Atomic similarities					
	gS	BN*	MN*	BN/MN*	First name	Date of birth
Pairs	38,083	38,083	38,083	76,166 **	38,083	38,083
Missing	0	0	31,747	57,088	0	0
Values at 1	9,566	25,990	5,348	3,065	26,017	26,505
in %	25.1	68.2	14.0	4.0	68.3	69.6

* BN = birth name, MN = married name

** Theoretical number of comparisons between BN and MN

**Table 2:** Characteristics of global similarity (gS) and atomic similarities (aS) in the identified pairs without exact concordance

Atomic similarities						
	gS	BN*	MN*	BN/MN*	First name	Date of birth
Pairs	28,517	12,093	988	16,013	12,066	11,578
Mean	0.92	0.79	0.78	0.36	0.77	0.82
Stand. error	0.48	0.20	0.23	0.25	0.17	0.07
Minimum	0.85	0.00	0.00	0.00	0.00	0.65
Percentiles						
25^th^	0.87	0.76	0.62	0.22	0.63	0.77
50^th^	0.92	0.88	0.90	0.41	0.82	0.85
75^th^	0.97	0.92	0.94	0.52	0.92	0.88
90^th^	0.99	0.95	0.96	0.63	0.95	0.88
95^th^	0.99	0.96	0.96	0.75	0.96	0.88

* BN = birth name, MN = married name

Among those 38,083 couples (**T**), 19,882 (**C**) may be found by classical techniques: first three last-name letters exact matching and first three first-name letters exact matching and date of birth matching and gender matching, while detection of the last 18,201 is made possible by using a string similarity algorithm. An in-depth analysis of those 18,201 paired records shows that 9,690 couples (**P**) are true positives, while 8,511 are false ones. Effectiveness over classical techniques can then be calculated: a minimum of P/(C+P) = 33% more true positive couples are detected than by a classical method (for reasons of convenience, the C couples are all declared to be true positives, while in fact, a few false positives may be present). The proportion of true positives according to this clerical review is shown on Figure 2, by different levels of global similarity (gS by unit of one). As a result, we can now assume that the positive predictive value defined as the number of true positives divided by the total number of linked pairs is (P+C)/T = 78%, which represents a good indicator of accuracy. In Figure 2, we see that the drops, at 0.96, 0.91 and 0.88, reflect name frequency adjustments.

Once the couples have been constituted and their global similarity values calculated, we can now define the relevant graphs. The 38,083 couples represent 27,184 clusters. This final step creates two kinds of clusters: 25,642 complete and 1,542 incomplete graphs. The first ones have all their UIDs (vertices) linked by an edge (global similarity above the threshold). The second ones present some UIDs that are not linked to some of the other cluster components. In the complete graphs there are 23,004 clusters of a size of 2 records, 2,237 of size 3 and 401 of a size greater than 3. In the incomplete graphs there are 1,012 clusters of size 3 and 530 of a size greater than 3. The complete graphs represent 54,443 initial distinct UIDs while the incomplete represent 5,760 UIDs. But before clerical review, our method allows us to state that the 25,642 complete graphs potentially represent 25,642 different individuals. Whatever the threshold within the range 0.85 to 1 is, the mean size for the graphs is constant; about 2.1 for complete graphs and 3.5 for incomplete graphs but the relative number of each kind of graph changes: 200-fold more complete graphs than incomplete for a threshold set to 1, 60-fold for 0.95 and 15-fold for 0.85. The use of the partition algorithm allows us to merge the 1,542 incomplete graphs into several complete graphs. The 1,012 incomplete clusters of size 3 become 1,012 single records and 1,012 couples. The other 530 incomplete graphs become 532 single records, 329 couples, 310 complete graphs of size 3 and 111 complete graphs of a size greater than 3. The 38,083 couples correspond to 60,203 initial UIDs. Out of a total number of UIDs of 300,859, 240,656 UIDs do not form part of a couple, corresponding to 240,656 different individuals. 25,642 individuals come from complete graphs and 3,306 from incomplete graphs. Finally, there are 269,604 different individuals in our database, corresponding to a 90% rate of uniqueness. This means that 1 record in 10 is involved in at least one cluster of "n-plicate" identities.

## Discussion

Our method gives those specialists responsible for merging similar records a representation showing how close records in some homogeneous sets are. But real-time detection is also one of the main goals of our proposal: avoiding duplicate entry by alerting the user that several neighbour records already exist, and furthermore, they help in decision making whilst providing proximity values between these similar records. This real-time use would be involved in multi-criteria searches for identities or for the creation of identities. But only a simple and easy to use front-end algorithm and short response time allow this possibility. Response times are closely related to an optimization of the algorithm and especially the blocking part. Its improvement allows the reduction in the number of potential duplicates to be tested by the main algorithm.

According to Porter and Winkler, the use of their algorithm improves the number of duplicates by 30% compared to a binary or semi-binary method [28]. Our results are in accordance with this number. Furthermore, we consider only the true positive identified couples in excess of those identified by the classical methods. Nevertheless, methods other than an approximate string matching can also be considered. For example, the CART method (classification tree-based models) could be used for mapping duplicates and defining clusters of records, which have to be seen not as duplicates but "n-plicates". A logistic model could also be proposed, using for its dependent variable a dummy variable indicating a duplicate or not, and the edit-distance as the independent variable. Some other independent variables can be also used.

Even if our approach is feasible and seems to be useful and reliable, we have to improve our process. The formal Jaro-Winkler algorithm is a domain-independent algorithm, which can be used, without any modification, for a wide range of applications. Our algorithm is no more domain-independent because of our weighting procedure of matching variables, and because of our names frequency based weighting. The classical approach in this context, to solve both problems, is to use the EM algorithm [9,10,34] to retrieve weights driven by the dataset. But the formalisation of using the EM algorithm was developed in the probabilistic Felligi-Sunter theory framework. Our approach is no longer within this framework since our weighted average of atomic similarity has no probabilistic interpretation. Furthermore, the conditional independence assumption of the components of the agreement vector is probably not fulfilled. One main basis of our approach is that all records do not have the same fields because of the potential absence of the married name. Hence the theoretical considerations for the extension of the EM algorithm to our approach are not so straightforward. Instead, other attractive methods seem to be machine-learning in a Bayesian classification framework [35,36]. The aim is to improve a partial automation of the linkage decision process, involving the use of a training data set, in order to instruct a learning algorithm to classify data in link or non-link. Moreover, our method essentially deals with real-time data browsing. In this issue the data set has to be viewed essentially as an evolving database and the machine learning seems to be very accurate: each decision taken by the user can become part of the learning process of the algorithm, instead of a new calculation of the weights by the EM algorithm.

The similarities we use (atomic and moreover global) do not fulfil the conditions necessary in order to qualify as a distance. An interesting condition on distance is the transitivity one, for example between records **A**, **B **and **C**: **d(A,B) **≤ **d(A,C) **+ **d(C,B)**. A weighted mean of several distances is indeed a distance unless the weights differ for at least one distance between records. This is the case in our approach (notably due to the difference of treatment between records with married name and records without) but that is also the case with the Porter-Jaro-Winkler algorithm due to the improvement involved when the four first characters of two strings are exactly matched: it can be the case between, for example, record **A **and **C **but not the case between records **B **and **C**. The condition of transitivity is not sufficient to be really useful in the case of string matching for sparing the number of comparisons between strings. A really interesting property would be the exact knowledge of the relation between **d(A,B)**, **d(A,C) **and **d(C,B)**. But this knowledge is probably not compatible with the complexity of building an indicator to evaluate string proximity and an indicator of neighbouring records. We noticed, empirically, that in our data set, the transitivity property seems to be fulfilled and hence increases the confidence it is possible to have in the Porter-Jaro-Winkler algorithm.

Here we use, *a posteriori*, an improvement of window algorithms already mentioned in literature [24,37,38]: records are ordered according to a given criterion and the algorithm, for a given record, is not used on the whole reference dataset, but only on records in a window of a given length. The standard method of detecting exact duplicates in a table is to sort the table and then to check if neighboring tuples are identical. Exact duplicates are guaranteed to be next to each other in the sorted order, regardless of which part of a record the sort is performed on. The idea is to do sorting to achieve preliminary clustering and then to do pairwise comparisons of nearby records. But in this case there is no guarantee that approximate duplicates are all next to each other in the sorted order. In the worst case, they will be found at opposite extremes of the sorted heap. In our case, the criterion is the blocking key and we used the algorithm on all the records sharing the same blocking keys. Much more than a sorted list, the canopy technique we use creates overlapping subsets in which records are compared. In the case of a dataset of size *n*, divided into *b *blocks by standard blocking or bigram indexing, the complexity of the record linkage decreases from O(*n*^2^) to O(*n*/*b*) but with a much larger *b *with bigram indexing. The complexity is O(*wn*) with the sorted neighbourhood with a *w*-size window. For canopy clustering, the number of record pair comparisons is O(*f*^2^*n*^2^/*c*) where *c *is the number of canopies and *f *the average number of canopies a record belongs to. In our data set, we find that the number of canopies has the same order as the number of records and that each record belongs to about 10 canopies (in mean). Our complexity decreases, then, from O(*n*^2^) to O(n), which is less accurate than a bigram indexing complexity. However, the calculation of canopies technique complexity over-estimates this complexity [26]. Actually, it seems to be clear that the most efficient improvement would be to consider not a window but a priority queue. For example Monge [20] proposes a three-step procedure. First, the Smith-Waterman algorithm is used to recognize pairs of approximate duplicates, then the union-find algorithm to keep track of clusters of duplicate records incrementally, as pairwise duplicate relationships are discovered. Thirdly, a priority queue of cluster subsets responds adaptively to the size and homogeneity of the clusters discovered as the database is scanned. All these improvements are nevertheless based on identification algorithms using edit-distance.

Our method adds to the calculation of similarity between couples, a step of clustering and partitioning. To our knowledge, no other published method in the field of record linkage offers these last steps. Hence, for comparing our method with the others, we have to rely on the similarity step even if this is not our "final product". The measurement of the quality of record linkage relies on 4 different values: the number of record pairs linked correctly (true positives), the number of record pairs linked incorrectly (false positives), the number of record pairs unlinked correctly (true negatives) and the number of record pairs unlinked incorrectly (false negatives). These values allow the calculation of different estimates of the performance of an algorithm and notably: the specificity (true negatives divided by the number of true non-match pairs), the negative predictive value (true negatives divided by the total number of non-linked pairs), the sensitivity (true positives divided by the total number of true match pairs, which is the sum of the true positives and the false negatives) and the positive predictive value (true positives divided by the total number of linked pairs). The goal of methods for approximate records matching is to retrieve the truest duplicates possible, even if the price to pay is to get some false positives as well. Thus, the interest is mostly in the sensitivity and in the positive predictive value of methods, corresponding with, respectively, the recall and the precision in the text retrieval field. Furthermore, the number of true negatives represents about 80 or 90 % of the total number of the pairs and any comparisons based on a quality indicator involving this number will be difficult to interpret. Indeed, for example, the specificity of different methods will always (except with bad methods) be close to 1 because the differences in the number of false positives will play a very minor role in the division by the number of true negatives. With respect to the tremendous number of records it is very time consuming, and almost impossible, to review all pairs non-linked by the methods we want to compare with, in order to detect false negatives and true negatives. The sensitivity (and the negative predictive value) is hence very difficult to calculate. Only the positive predictive value is a reliable and easy to calculate indicator for measuring the performance of a method for record linkage. As it is quite impossible to review all the pairs, a solution would be to sample some pairs and just review these ones. But this solution does not seem to be completely accurate as the main quality of sampling is to respect the "data generating process". In the case of duplicate identities generating, this process seems to be much too complicated to be reproduced. The risk then, is to have a biased validation sample and to get a wrong quality indicator. Another solution is to have an efficient external data set with several known characteristics. The underlying idea is to compare an exhaustive and clean (meaning without duplicates, after clerical review) data set with these characteristics, with the subset of the general hospital data set with these same characteristics. This procedure was, for example, (with people remaining anonymous) used with a digestive cancer registry [39].

The most common used final indicator, in the framework of record matching, is the percentage of duplicate identities. But we cannot rely on this indicator as our method builds several clusters of different sizes; each cluster corresponding, after clerical review, to one identity. Hence we use an indicator of uniqueness in the database calculated as the sum of the number of UIDs not involved in any cluster and the number of clusters, divided by the number of initial UIDs. As some clusters are not size 2, this uniqueness is not directly related to the number of duplicate identities (meaning couples whose similarity is above the threshold).

When we identify clusters of n-plicates, we have to decide, furthermore, whether they are real n-plicates. The last step is then to merge, not only on the identities – which is already a problem – but also the medical records relating to these identities. Even if all the global similarities are 1 in a given cluster, identified n-plicates stay "possible n-plicates" and only a very close examination can allow us to discard homonymic identities (in our case, same names, same first name, same date of birth, same gender and same address but different subjects). If merging clients' addresses is not so risky, the problem is quite different in the case of medical records. Complete traceability should be possible for "un-merging" clusters if necessary. The necessity of a human decision for merging is the most important limitation for a completely automatic process.

Using matching variables other than the minimum set we defined would dramatically improve the efficiency of the records matching. For example, parents name or the city of birth is a very discriminatory matching variable. Furthermore, the first name seems to be less reliable than the birth name for comparing records, and difficulties with married names yield a complex algorithm for the calculation of global similarity. Hence, perhaps the minimal set for identifying individuals is neither really reliable nor sufficient.

## Appendix

The goal of this section is to detail the weighting procedure of the atomic similarities for building the global similarity as a weighted mean of those same atomic similarities. The Table 3 summarizes this procedure. We call **A **the first record (reference record) and **B **the second record (sample record). Each record contains four different fields (birth name, married name, first name and date of birth) whose value cannot be the null string (noted **{0}**) unless it is the married name. These fields, for example in record **A**, are respectively noted **A(BN)**, **A(MN)**, **A(FN) **and **A(DB)**. We call **aS **the vector of atomic similarities and **gS **the global similarity. The components of **aS **are the atomic similarities between two fields: which are noted **aS(BN) **between the two birth names, **aS(MN) **between the married names, **aS(BN/MN) **between the birth name of the first record and the married name of the second, **aS(MN/BN) **between the married name of the first record and the birth name of the second, **aS(FN) **between the first names and **aS(DB) **between the dates of birth. **T **is the threshold under which two atomic similarities are acknowledged to correspond to two different values of the fields. With reference to Porter-Jaro-Winkler, this threshold is fixed to 0.7. In the procedure there are four main cases, each one is further divided in several sub-cases.

**Table 3:** Weighting procedure of the atomic similarities (Appendix)

Weights	**BN**	**MN**	**MN/BN**	**BN/MN**	**FN**	**DB**
Case 1						
**A(MN) = {0} **and **B(MN) = {0} **then						
If **aS (BN) <T **or **aS (FN) <T **then	2/4	-	-	-	1/4	1/4
Else	2/6	-	-	-	1/6	3/6

Case 2						
**A(MN)! = {0} **and **B(MN) = {0}**						
**(1) **If **aS (MN/BN) <aS (BN) **then						
If **aS (BN) <T **or **aS (FN) <T **then	2/4	-	-	-	1/4	1/4
Else	2/6	-	-	-	1/6	3/6
(*) Else	1/6	-	1/6	-	1/6	3/6

Case 3						
**B(MN)! = {0} **and **A(MN) = {0}**, switch **A **and **B **and go to **(1)**						

Case 4						
**A(MN)! = {0} **and **B(MN)! = {0}**						
If **aS (BN/MN) <aS (BN) **and **aS (MN/BN) <aS (MN) **then	2/7	1/7	-	-	1/7	3/7
If **aS (BN/MN) >aS (BN) **and **aS (MN/BN) <aS (MN) **then	1/7	1/7	-	1/7	1/7	3/7
If **aS (BN/MN) < aS(BN) **and **aS (MN/BN) >aS (MN) **then	1/7	1/7	1/7	-	1/7	3/7
If **aS (BN/MN) >aS (BN) **and **aS (MN/BN) >aS (MN) **then	1/8	1/8	1/8	1/8	1/8	3/8

For example, suppose that the atomic similarities for two records **A **and **B **are **aS(BN) = 0.80**, **aS(MN/BN) = 0.97**, **aS(FN) = 0.90 **and **aS(DB) = 0.92**. This case corresponds to a first record with a married name and a second record without a married name but with a birth name which is nearer the married name of the first record than the birth name of this first record (probably an inversion between the two names). In the Table 3, these atomic similarities go with the row highlighted with (*). Hence, the global similarity is calculated as **1/6.0.80 + 1/6.0.97 + 1/6.0.90 + 3/6.0.92 **or **0.905**. Even if there is a further error with the dates of birth, the global similarity indicates a relatively strong proximity between the two records. Of course, if the errors on dates of birth were more pronounced, the global similarity would quickly decrease.

## Competing interests

The author(s) declare that they have no competing interests.

## Authors' contributions

EAS reviewed papers on the subject and built, with JPP, the methodology. JPP is in charge of all methods implementation. AB supervised this work. All authors read and improved successive drafts.

## Pre-publication history

The pre-publication history for this paper can be accessed here:


